# Calcium Supplement Combined with Dietary Supplement Kidtal Can Promote Longitudinal Growth of Long Bone in Calcium-Deficient Adolescent Rats

**DOI:** 10.3390/nu17121966

**Published:** 2025-06-10

**Authors:** Haosheng Xie, Mingxuan Zhang, Zhengyuan Zhou, Hongyang Guan, Kunmei Shan, Shiwei Mi, Xinfa Ye, Zhihui Liu, Jun Yin, Na Han

**Affiliations:** 1 Development and Utilization Key Laboratory of Northeast Plant Materials, School of Traditional Chinese Materia, Shenyang Pharmaceutical University, Wenhua Road 103, Shenyang 110016, China; xhs12315@163.com (H.X.); gudghdjvdhvdh@gmail.com (M.Z.); fa52xaw52x@163.com (H.G.); 15846698649@163.com (K.S.); 18581040387@189.cn (S.M.); 18563978930@163.com (X.Y.); liuzhihuishenyang@163.com (Z.L.); 2 Inne Institude of Nutrition and Health, Bismarckstraße 37, 66121 Saarbrücken, Germany; zzypai@163.com (Z.Z.)

**Keywords:** bamboo shoot extract, growth retardation, amino acids, calcium, endochondral ossification, GH-IGF axis

## Abstract

**Objective:** Growth retardation in adolescents caused by nutritional deficiency requires effective intervention. A novel dietary supplement containing bamboo shoot extract, amino acids, and calcium citrate (Kidtal + Ca, KDTCa) was evaluated for its growth-promoting effects. **Methods:** After acclimatization, sixty-three 3-week-old male Sprague-Dawley (SD) rats were randomly divided into a normal control group and model groups. Growth retardation was induced in the modeling groups through calcium-deficient feeding, followed by administration of KDTCa, bamboo shoot extract and amino acids (Kidtal), or calcium citrate (CC). After 6 weeks of intragastric administration, the mechanical properties, microstructure, and growth plate development of bone were evaluated using three-point bending, micro-CT, and H&E staining, respectively. Bone calcium/phosphorus distribution and fecal calcium apparent absorption rate were measured by ICP-MS. **Results:** All inter-group differences were analyzed using one-way analysis of variance and checked using the Tuckey test. KDTCa treatment dose-dependently enhanced bone development in calcium-deficient rats. Compared to the model group, H-KDTCa significantly restored naso-anal length (*p* < 0.05) and body weight (*p* < 0.01). KDTCa supplementation significantly restored calcium and phosphorus levels in blood and bone. Three-point bending experiments showed that the stiffness and bending energy were increased by 142.58% and 384.7%. In bone microarchitecture, both bone mineral density (BMD) and microstructural parameters were significantly improved. These findings were consistent with the increased long bone length (*p* < 0.05) and decreased serum BALP/TRACP levels (*p* < 0.001). Dose-dependent IGF-1 elevation (*p* < 0.01) potentially mediated growth plate elongation by 35.34%. Notably, KDTCa increased calcium apparent absorption by 6.1% versus calcium-only supplementation at equal intake. **Conclusions:** KDTCa improves bone microstructure and strength, restores bone metabolism, and enhances growth plate height via promoting IGF-1 secretion to facilitate bone development. Further studies are needed to determine whether the components and calcium in Kidtal have a synergistic effect.

## 1. Introduction

Height growth is a critical physiological process in children’s development, influenced by various factors such as genetics, nutrition, hormones, and environmental conditions [1]. Adolescent growth retardation is a common developmental issue that can lead to stunted height [2], delayed bone development, and overall slow growth. It not only affects the physical health of adolescents but may also lead to psychological issues, including low self-esteem, social difficulties, and an increased risk of other diseases in the future [3,4].

Current treatments for adolescent growth retardation primarily focus on nutritional supplementation and growth hormone (GH) therapy [5,6]. GH is one of the most commonly used clinical treatments for short stature. It promotes longitudinal bone growth by stimulating the proliferation and maturation of chondrocytes through Insulin-like Growth Factor 1 (IGF-1) [7]. Although GH therapy effectively promotes bone growth, it also has significant limitations, including high costs, a potential increased risk of cancer, altered blood glucose levels, and side effects such as premature puberty [8]. Therefore, there is an urgent need for a safe, effective, and economically viable alternative treatment to address adolescent growth retardation.

Malnutrition is a key factor that limits children’s growth [9]. An adequate intake of essential nutrients, such as calcium, phosphorus, and vitamin D, is crucial for bone mineralization and maintaining bone health. [10]. Common calcium supplements, including both organic and inorganic forms, present certain drawbacks [11,12]. Therefore, vitamin D and calcium supplements are often used together to promote calcium absorption. However, some studies have found that long-term intake of vitamin D may potentially lead to hypercalcemia, resulting in calcium–phosphorus imbalance [13].

Amino acids, as essential nutrients, serve as raw materials for sphingolipid and glycerophospholipid synthesis and play a crucial role in childhood development. Compared to adults, children have a higher amino acid requirement [14,15,16]. Natural amino acids remain the primary source of amino acids for the human body, providing greater potential for growth and development. Multiple amino acids, such as arginine and lysine, have been demonstrated to exert positive effects on growth and development; as an amino acid and a neurotransmitter, GABA has been shown to have an effect on bone health [17,18,19,20].

Bamboo shoots, which are from the tender buds of bamboo, have been consumed in China for approximately 3000 years as medicinal food homology plants [21]. They are credited with the properties of strengthening the stomach and promoting bowel regularity in ancient writings. Modern studies have further demonstrated that bamboo shoots are rich in proteins, amino acids, cellulose, and various other nutrients [22] and may play a key role in growth and development. Although modern research on bamboo shoots is abundant, studies regarding their impact on growth and development remain limited. Therefore, as a natural plant with substantial global market potential, bamboo shoots warrant further research and development regarding their osteogenic activity.

KDTCa is a combined calcium supplement that is composed of calcium citrate, L-lysine hydrochloride, L-arginine, γ-aminobutyric acid, and bamboo shoot extract. Previous research has demonstrated that it played growth-promoting effects in zebrafish. To further investigate its potential functions and the effect of KDT in this combination, they were applied to an adolescent model with growth impairment induced by a low-calcium diet, aiming to explore a safer and more effective approach to promote growth and development.

## 2. Materials and Methods

### 2.1. Materials

The calcium citrate was bought from Yunbo Health Technology Co., Ltd. (Qinzhou, China), the L-arginine, L-lysine hydrochloride, and bamboo shoot extract were provided by Beijiguang Biotechnology Co., Ltd. (Nanjing, China), and γ-aminobutyric acid (GABA) was obtained from Letop Biotechnology Co., Ltd. (Nanjing, China). The serum calcium and phosphorus reagent kits, as well as the serum IGF-1 ELISA kit, were purchased from Jianglai Industry Co., Ltd. (Shanghai, China). The serum TRACP and BALP kits were obtained from Baililai Biotechnology Co., Ltd. (Shanghai, China). All feed types were sourced from Nantong Teluofei Feed Technology Co., Ltd. (Nantong, China).

### 2.2. Sample Preparation

Calcium citrate, L-lysine hydrochloride, L-arginine, γ-aminobutyric acid, and bamboo shoot extract were mixed thoroughly at a mass ratio of 274.3:48:9.6:9.6:2.2 for the preparation of the calcium combination (KDTCa). A calcium-free combination (KDT) was composed with L-lysine hydrochloride, L-arginine, γ-aminobutyric acid (GABA), and bamboo shoot extract at a mass ratio of 48:9.6:9.6:2.2. KDTCa, KDT, and calcium citrate (CC) were suspended ultrasonically in 0.5% CMC-Na solution for administration to animals.

### 2.3. Animal Modeling and Drug Delivery

This study was conducted in strict compliance with the ethical guidelines and regulations of the Experimental Animal Center of Shenyang Pharmaceutical University (Shenyang, China). All experimental procedures were reviewed and approved by the Experimental Animal Research Committee of Shenyang Pharmaceutical University (SYPU-ZACUC-S2024-0717-204). This experiment used 3-week-old male SD rats (ChangSheng Biotechnology Co., Ltd., Benxi, China). All animals were housed in a specific pathogen-free (SPF) environment (22 ± 2 °C, 60 ± 5% relative humidity, and 12/12 h light/dark alternate cycles). During the experimental period, all animals had free access to deionized water and food.

**Figure 1:**
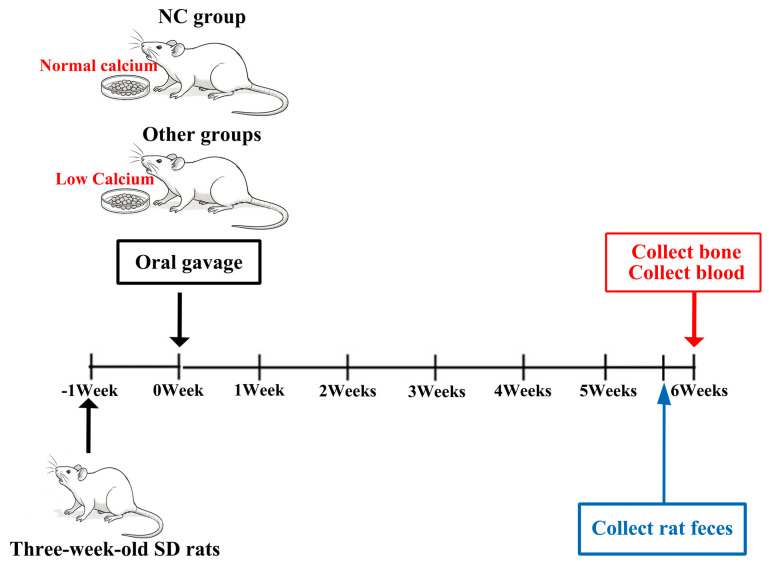
Graphical representation of the animal experiment.

After a one-week acclimatization period, 63 SD rats were randomly allocated by body weight into seven groups (*n* = 9) using a random number table. The groups were as follows: a normal-calcium control group (NC group), low-calcium model group (LCM group), calcium citrate treatment group (CC group, 274.3 mg/kg calcium citrate), low-dose KDTCa group (L-KDTCa group, 343.7 mg/kg test sample), medium-dose KDTCa group (M-KDTCa group, 687.4 mg/kg test sample), high-dose KDTCa group (H-KDTCa group, 1374.8 mg/kg test sample), and bamboo shoot and amino acids combination group (KDT group, 69.4 mg/kg KDT). The NC group was provided with AIN-93 standard feed containing 0.5% calcium [23], while all other groups were fed a low-calcium diet with a calcium content of only 0.004%. The treatment was administered once daily via gavage at a dose of 1 mL/kg body weight for six weeks and the experimental unit was each rat. Body weight and nose–tail length was measured weekly (Figure 1).

### 2.4. Blood Sample Collection

Before the experiment concluded, each rat was placed in an individual metabolic cage for a three-day fecal collection period. After six weeks of gavage, the rats were fasted overnight. Then, all animals were anesthetized with sodium pentobarbital. After blood collection from the abdominal aorta in live rats, the animals were euthanized by cervical dislocation. The blood was left to stand at room temperature for 2 h and then centrifuged at 2000× *g* for 20 min to separate serum. The serum was retained at −80 °C.

### 2.5. Bone Sample Collection

The hind limbs of the rats were dissected, the femurs and tibias were obtained after removing the surrounding muscle tissue and rinsing three times with deionized water, followed by three rinses with physiological saline, and the lengths of two types of long bones were determined using a digital caliper. For each group, five right femurs and all right tibias were fixed in 4% paraformaldehyde, while the remaining bones were wrapped in saline-soaked gauze and stored at −80 °C.

### 2.6. Measurement of Serum Bone Metabolism Markers and Mineral Content

The serum was aliquoted into four portions and stored at −80 °C, with one aliquot thawed at room temperature for each ELISA assay. Serum levels of Tartrate-Resistant Acid Phosphatase (TRACP), Bone-Specific Alkaline Phosphatase (BALP), and Insulin-like Growth Factor 1 (IGF-1) were determined using an enzyme-linked immunosorbent assay (ELISA) kit. The serum calcium and phosphorus levels were measured using rat serum calcium and serum phosphorus assay kits.

### 2.7. Bone Biomechanical Parameter Measurement

The three-point bending test was performed on right femurs using an INSTRON 3366 universal testing machine (INSTRON, Norwood, MA, USA). The rat femur was removed from the −80 °C freezer and thawed at room temperature in physiological saline. After thawing, the femur was placed on two supporting points of a universal testing machine and the test was conducted with a support span of 20 mm, a loading head diameter of 4 mm, and a loading rate of 2 mm/min, continuing until the femurs fractured. The experimental parameters were automatically recorded by computer and the stiffness and fracture energy were determined from the load–displacement curve.

### 2.8. Micro-Computed Tomography (CT) Analysis

The right femurs were fixed in 4% paraformaldehyde for 2 days, then washed 10 min with deionized water, followed by another three rinses with physiological saline. Bone microstructure was analyzed using a VNC-102 Micro-CT system (Venus, Kunshan, China) under the following parameters: voltage of 90 kV, current of 0.09 mA, and a scanning resolution of 30 μ m. Data acquisition was performed using Cruiser software and 3D image reconstruction was carried out using Recon software. The measured parameters included bone mineral density (BMD), bone volume (BV), trabecular bone volume fraction (BV/TV), trabecular thickness (Tb.Th), trabecular number (Tb.N), and trabecular separation (Tb.Sp).

### 2.9. Histological Analysis

The rat tibiae were initially fixed in 4% paraformaldehyde for 2 days, then decalcified in a 10% EDTA solution for 42 days, with the solution being refreshed weekly. Once decalcification was complete, the samples were embedded, sectioned, and processed for hematoxylin and eosin (H&E) staining.

The method described by Lee [24] was slightly modified to measure the growth plate of the proximal tibia. The total growth plate height, along with the heights of the proliferative and hypertrophic zones, was measured at six different locations using ImageJ 2.14.0 software and the mean values were calculated. These measurements were independently performed by three experienced researchers.

The vertical height of the resting zone was calculated as follows:

Resting Zone Height = Total Growth Plate Height − (Proliferative Zone Height + Hypertrophic Zone Height)(1)

### 2.10. Elemental Analysis by Inductively Coupled Plasma Mass Spectrometry (ICP-MS)

The calcium and phosphorus contents in the rat femur and feces were measured using previously reported methods and the element content of bamboo shoot extract [25]. The femurs and feces were dried in an oven at 105 °C until the weight no longer changed. After this step, we recorded the dry weight of the femurs. The three types of samples were then transferred to crucibles, treated with 1 mL of nitric acid, and heated on an electric furnace until carbonization was complete and no smoke remained. Following this step, the ashing process was carried out in a muffle furnace at 500 °C for 4 h.

The two elements were analyzed using ICP-MS. The apparent calcium absorption rate was calculated as follows:

Calcium apparent absorption rate (%) = [(Intake Calcium − Fecal Calcium)/Intake Calcium] × 100%.(2)

### 2.11. Determination of Hydrolyzed Amino Acids in Bamboo Shoot Extracts

Following a modified version of the method described by Xu et al. [26] an appropriate amount of bamboo shoot extract powder was weighed. Subsequently, 10 mL of analytical-grade hydrochloric acid (1:1, approximately 6 M) was added and nitrogen gas was introduced into the tube for 30 s to expel oxygen. The sealed tube was then subjected to hydrolysis in an oil bath maintained at 110 °C for 22 h. Then, the resulting solution was filtered through a 0.45 μm membrane into a 50 mL volumetric flask and diluted to the desired volume. A 2 mL aliquot of this diluted solution was taken and heated at 85 °C twice to remove residual acid. Thereafter, 1 mL of sodium citrate buffer was added and thoroughly mixed until completely dissolved. The final solution was passed through a 0.22 μm filter before analysis using a Biochrom 30+ Amino Acid Analyzer (Biochrom, Cambridge, UK).

### 2.12. Analysis of Nutritional Components of Bamboo Shoot Extract

The total polysaccharide content in the bamboo shoot extract was determined using glucose as a standard with the sulfuric acid–acetone method, while the protein content was measured by Bradford assay using bovine serum albumin (BSA) as the standard.

### 2.13. Statistical Analysis

The experimental results were expressed as mean ± standard deviation (SD). Normality was assessed using the Shapiro–Wilk test and homogeneity of variance was evaluated using the Bartlett test in GraphPad Prism (version 10.1.2; GraphPad Software, San Diego, CA, USA). One-way analysis of variance (ANOVA) followed by Dunnett’s test was performed if assumptions were met; otherwise, Welch’s ANOVA was applied. Statistical significance was set at *p* < 0.05.

## 3. Results

### 3.1. Effects of KDTCA on Body Weight and Length in Calcium-Deficient Rats

**Figure 2:**
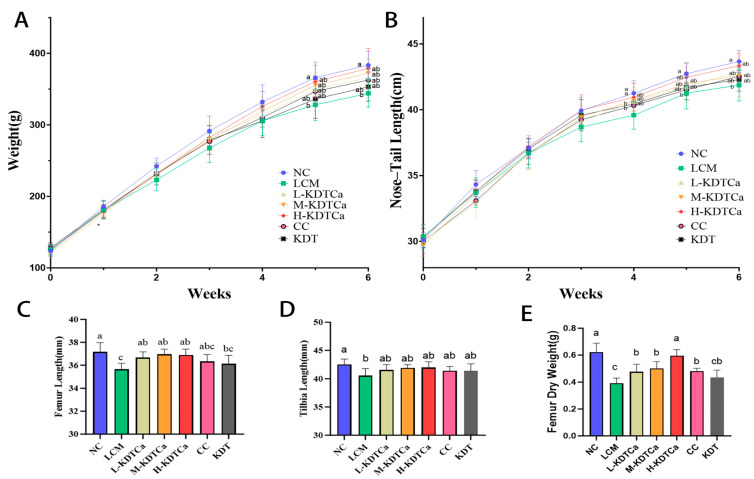
Effect of KDTCA on body parameters in rats (*n* = 9). (**A**) Body weight; (**B**) nose–tail length; (**C**) femur length; (**D**) tibia length; and (**E**) femur dry weight. Shared letters indicate non-significant group comparisons (*p* > 0.05), whereas distinct letters mark significant differences.

After feeding with a low-calcium diet, the body weight and nose–tail length in the LCM group showed a significant decrease compared to the NC group (*p* < 0.05) (Figure 2A,B). However, continuous KDTCa treatment mitigated this effect, as rats in the KDTCa groups slightly increased their body weight and nose–tail length compared to the LCM group by the 5th week (*p* < 0.05).

The length of long bones is a critical determinant of growth. We further measured the femur and tibia lengths. As shown in Figure 2C–E, bone development in the LCM group was impaired after calcium deficiency. KDTCa treatment significantly restored the bone growth defects caused by calcium deficiency. However, the administration of CC did not show obvious curative effects on bone lengths at the same calcium dose as in the L-KDTCa group, suggesting that the combination might have more advantages in promoting bone growth. The bone dry weight (Figure 2E), which partially reflects bone health status, showed significant increases (*p* < 0.01) in all calcium-supplemented treatment groups. Treatment with KDT alone did not result in any therapeutic benefits, indicating that calcium supplementation in the combination still played a crucial role in long bone development.

### 3.2. Biochemical Markers of Bone Metabolism

**Figure 3:**
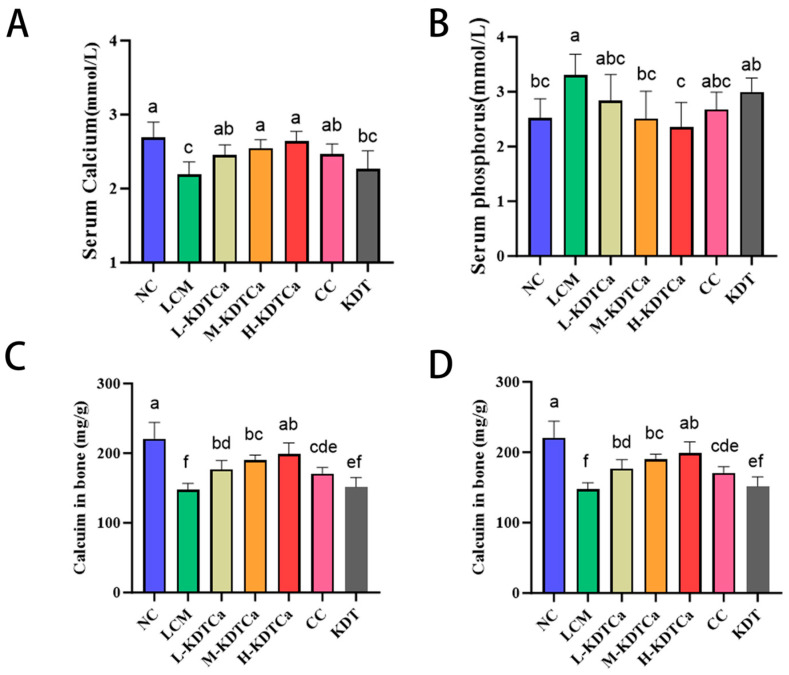
Effect of calcium and phosphorus content in serum and femur (*n* = 9). (**A**) Serum calcium; (**B**) serum phosphorus; (**C**) calcium in femur; and (**D**) phosphorus in femur. Shared letters indicate non-significant group comparisons (*p* > 0.05), whereas distinct letters mark significant differences.

Serum calcium and phosphorus levels are closely associated with bone health and serve as indicators of bone metabolism. In Figure 3A,B, the changes in serum calcium and phosphorus levels in rats are presented. The results indicate that serum calcium levels in the LM group were significantly reduced (*p* < 0.001), while serum phosphorus levels were significantly elevated (*p* < 0.05). After treatment, serum calcium levels significantly increased in all treatment groups except the KDT group (Figure 3A). The phosphorus levels in the M-KDTCa and H-KDTCa groups showed a significant decrease, approaching those of the NC group (Figure 3B).

As shown in Figure 3C,D, bone calcium (*p* < 0.001) and phosphorus (*p* < 0.001) content in the LCM group were significantly reduced. Following oral treatment, bone calcium and phosphorus content were significantly restored in the four treatment groups. Additionally, in the three KDTCa groups, the levels showed a dose-response relationship. However, there was no recovery in the KDT group.

### 3.3. Analysis of Serum Biochemical Indices

**Figure 4:**
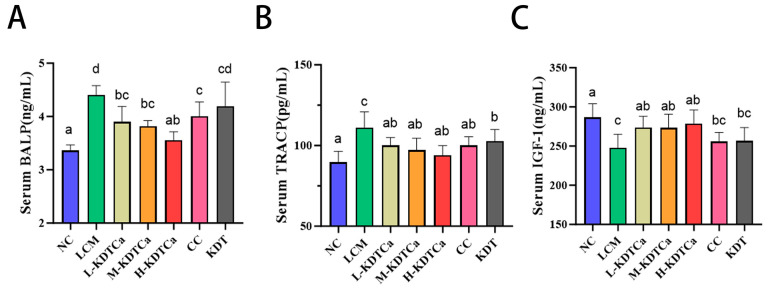
Effects on serum biomarkers (*n* = 9) (**A**) BALP; (**B**) TRACP; and (**C**) IGF-1. Shared letters indicate non-significant group comparisons (*p* > 0.05), whereas distinct letters mark significant differences.

The changes in serum biomarkers are illustrated in Figure 4. Osteoporosis markers BALP and TRACP significantly increased following the low-calcium diet (*p* < 0.01), whereas their levels significantly decreased in the four treatment groups (*p* < 0.05) in a dose-dependent manner (Figure 4A,B). Additionally, all three concentrations of KDTCa treatment significantly restored the decline in IGF-1 caused by prolonged low calcium intake (*p* < 0.05). No such effect was observed when CC or KDT was administered alone (*p* > 0.05) (Figure 4C), suggesting that the combined ingredients have a unique advantage in promoting IGF-1 secretion compared to a single calcium supplement.

### 3.4. Analysis of Bone Biomechanical Indexes

**Figure 5:**
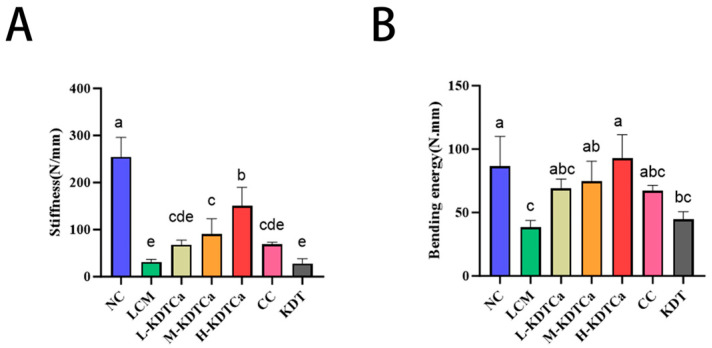
Effects on bone biomechanical parameters (*n* = 4). (**A**) Stiffness; (**B**) bending energy. Shared letters indicate non-significant group comparisons (*p* > 0.05), whereas distinct letters mark significant differences.

In Figure 5, the stiffness and bending energy of the femur in rats are displayed. The bone biomechanical properties in the LCM group significantly declined (*p* < 0.001). After treatment, femoral stiffness was significantly restored in the M-KDTCa and H-KDTCa groups. Increases of 118.99% and 122.72% were observed in the L-KDTCa and CC groups, respectively, compared to the LCM group, though these changes were not statistically significant (Figure 5A). After gavage treatment, the bending energy of all treatment groups, except for the KDT group, increased significantly, highlighting the critical role of calcium in the combination (Figure 5B).

### 3.5. Bone Microstructural Analysis

**Figure 6:**
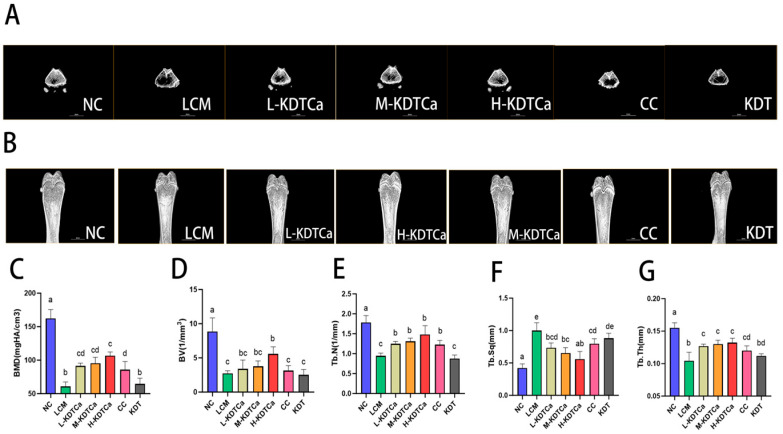
Effect of KDTCA on body parameters in rats (*n* = 5). (**A**) 3D cross-sectional image of the femur; (**B**) 3D reconstructed image of the femur; (**C**) BMD; (**D**) BV; (**E**) Tb.N; (**F**) Tb.Sq; and (**G**) Tb.Th. Shared letters indicate non-significant group comparisons (*p* > 0.05), whereas distinct letters mark significant differences.

Micro-CT 3D images show severe disruption of bone microarchitecture in the LCM group (Figure 6A,B), accompanied by reduced bone mineral density (BMD) and significant declines in all bone microstructural parameters (*p* < 0.001). As shown in Figure 6D, bone volume (BV) showed a recovery trend in all four treatment groups, except for the KDT group, although only the H-KDTCa group exhibited a significant improvement (*p* < 0.01). Moreover, the BMD (Figure 6C), trabecular number (Tb.N) (Figure 6E), trabecular separation (Tb.Sp) (Figure 6F), and trabecular thickness (Tb.Th) (Figure 6G) were significantly restored in all calcium-containing treatment groups, resulting in denser and more complete trabeculae, indicating effective restoration of bone microarchitecture (Figure 6).

### 3.6. Effects on the Growth Plate of Tibial

**Figure 7:**
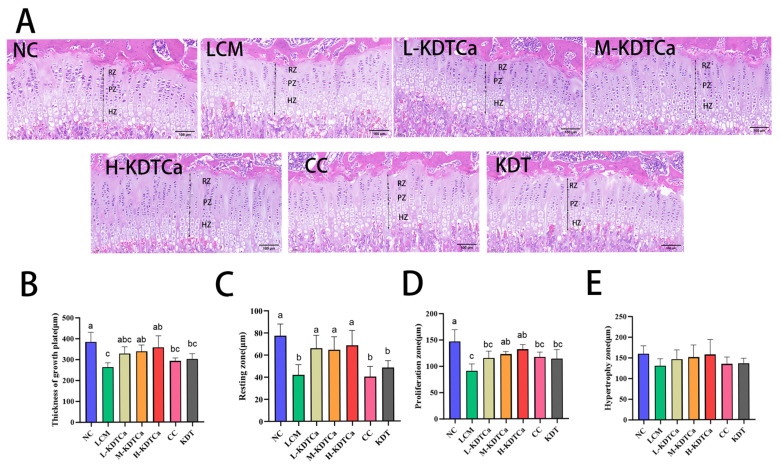
Effects of KDTCA on different regions of the growth plate (*n* = 5). (**A**) Growth plate histological sections (RZ: resting zone, PZ: proliferative zone, HZ: hypertrophic zone); (**B**) total growth plate height; (**C**) height of the resting zone; (**D**) height of the proliferative zone; and (**E**) height of the hypertrophic zone. Shared letters indicate non-significant group comparisons (*p* > 0.05), whereas distinct letters mark significant differences.

Histological analysis of tibial growth plates through HE staining demonstrated a significant reduction in the total growth plate height (Figure 7A), as well as in the heights of the resting and proliferative zones, in the LCM group compared to the NC group (*p* < 0.01). A slight decrease was also observed in hypertrophic zone height (Figure 7E). Notably, KDTCa treatment successfully counteracted this trend, resulting in a significant increase in the total height of the growth plate, as well as the heights of the resting and proliferative zones, across all three KDTCa dosage groups (Figure 7B–D). In contrast, the CC group exhibited only a slight increase in proliferative zone cell numbers (Figure 7C), with no substantial therapeutic effects detected in other parameters. No therapeutic effect was observed in the KDT group.

### 3.7. Effects of Calcium Apparent Absorption Rate

**Figure 8:**
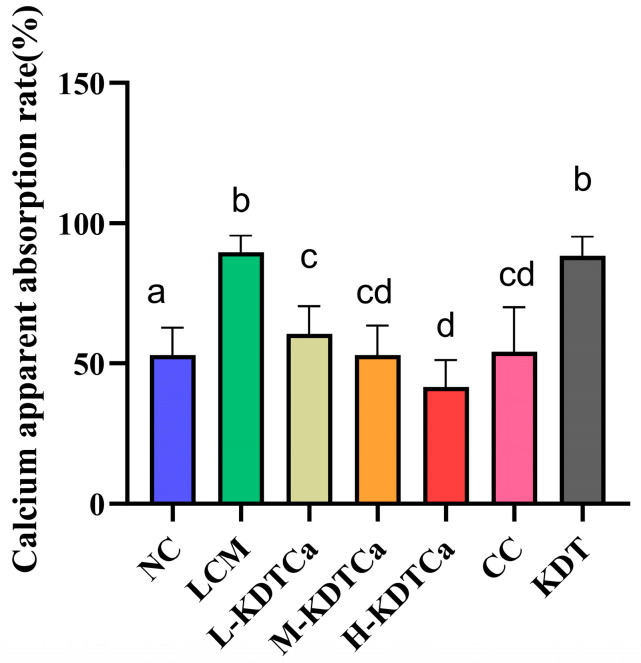
The effect of KDTCA on apparent calcium absorption rate (*n* = 9). Shared letters indicate non-significant group comparisons (*p* > 0.05), whereas distinct letters mark significant differences.

The apparent calcium absorption rate can assess the body’s efficiency in utilizing calcium. Rats fed with a low-calcium diet exhibited a significantly higher apparent calcium absorption rate than other groups (Figure 8), likely due to their extremely low calcium intake. Moreover, when calcium intake was the same, the absorption rate in the L-KDTCa group (60.5 ± 9.878%) was slightly higher than that observed in the CC group (54.1 ± 15.98%). With increasing calcium intake, the apparent absorption rate in the three KDTCa treatment groups decreased in a dose-dependent manner.

### 3.8. Nutrient Analysis of Bamboo Shoot Extract

**Figure 9:**
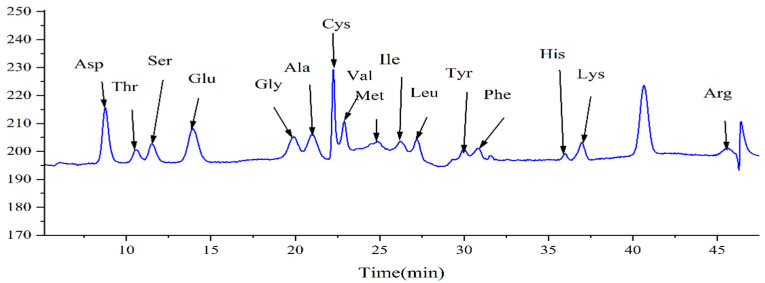
The chromatogram was obtained from the analysis of bamboo shoot extract using an automated amino acid analyzer.

As shown in Figure 9, 16 amino acids (including Asp, Thr, Ser, Glu, Gly, Ala, Cys, Val, Met, Ile, Leu, Tyr, Phe, His, Lys, and Arg) were detected at 570 nm after the hydrolysis of the bamboo shoot extract. The content of each amino acid is presented in Table 1. The total amino acid content in the bamboo shoot extract was 33.968 mg/g, with the two highest concentrations being Asp and Glu, at 6.283 mg/g and 6.218 mg/g, respectively.

The protein and polysaccharide contents in the bamboo shoot extract were determined using the Bradford method and the sulfuric acid–acetone method, respectively. The results showed that it contained 4.661% protein and 27.28% polysaccharides (Table 2). Furthermore, bamboo shoot extract contains various beneficial trace elements, such as potassium, sodium, iron, calcium, and zinc (Table 3).

## 4. Discussion

In this research, a dietary supplement combination containing bamboo shoot extract was evaluated for its effects on the growth and development of long bones in adolescent rats, with an experimental period of 6 weeks. Calcium plays a crucial role in the growth and development of rats, and insufficient intake leads to delayed growth [27,28]. Rats in the model group showed growth retardation, likely as a result of prolonged calcium deficiency (Figure 2).

Calcium and phosphorus are the two most abundant metallic elements in bone. Calcium and phosphorus levels in serum and bone are closely associated with bone health and serve as indicators of bone metabolism [29]. Due to prolonged calcium deficiency, serum calcium levels decreased in rats fed a low-calcium diet (Figure 3A,B), which is consistent with the results of Peng et al. [30]. This may be due to a negative calcium balance resulting from prolonged low-calcium intake, which is often associated with decreased serum calcium levels and increased parathyroid hormone levels, promoting the release of calcium and phosphorus from bones into the bloodstream [31,32]. However, due to the low Ca/P ratio in the calcium-deficient diet, calcium deficiency in the bone resulted in the absence of calcium that could be released into the bloodstream, while phosphorus continued to be released into the blood, ultimately leading to decreased blood calcium and increased blood phosphorus. As anticipated, KDTCa treatment effectively reversed this phenomenon, attributable to the combined mineral supplementation from calcium and bamboo shoot extract (Figure 3A,B).

The aforementioned phenomena can be further validated by observing changes in the levels of BALP and TRACP [33,34]. Elevated BALP levels indicate accelerated bone turnover, whereas increased TRACP levels signify excessive osteoclast-mediated bone resorption [35]. As we expected, KDTCa treatment significantly inhibited bone resorption (Figure 4A,B), thereby leading to a remarkable recovery of bone mineral levels (Figure 3). These findings demonstrate that KDTCa treatment inhibits bone resorption and is more effective than calcium citrate alone.

The biomechanical characteristics of bone, particularly its strength, can be evaluated through parameters such as stiffness and bending energy, which serve as critical indicators of its mechanical integrity [36,37]. Both CC and KDTCa significantly restored bone biomechanical parameters, while KDT treatment had no such effect. We hypothesize that calcium supplementation, rather than the other components of KDTCa, is the primary factor responsible for restoring bone mechanical strength (Figure 5). To further explore the reasons behind the improvement in femoral biomechanical parameters, we performed micro-CT analysis of the bone microstructure. We found that after gavage treatment, the bone microstructure of rats was more intact, indicating that KDTCa may enhance bone strength by improving bone density and restoring trabecular structure (Figure 6C,E–G). Furthermore, compared to calcium citrate at the same dose, the L-KDTCa group exhibited a better recovery trend in Tb.N, Tb.Sp, and Tb.Th (*p* > 0.05), suggesting that the amino acids and bamboo shoot extract in KDTCa demonstrate enhanced therapeutic potential for trabecular microstructure, indicating a synergistic interaction with calcium (Figure 6E–G). Hu et al. demonstrated that the combined administration of sheep bone protein hydrolysates (SBPHs) and CaCl_2_ significantly enhanced growth rate and restored bone microstructure in calcium-deficient rats, with superior effects compared to CaCl_2_ alone. We believe that the superior efficacy of KDTCa compared to calcium citrate may be attributed to the inclusion of KDT, which, like SBPHs, is likely rich in proteins and amino acids. This observed enhancement may be mediated through improved calcium binding and absorption facilitated by amino acid residues present in the bamboo shoot extract, although further experimental validation is required to confirm this mechanism.

IGF-1 is a key component of the growth hormone (GH)–Insulin-like Growth Factor (IGF) axis and facilitates growth by activating the Akt and ERK signaling pathways, playing a vital role in bone development [38]. Compared to the LCM group, serum IGF-1 levels were significantly elevated in all three KDTCa treatment groups but not in the CC and KDT groups, a trend that aligns with the measured femoral and tibial lengths (Figure 4C). Notably, individual administration of these components fails to significantly elevate IGF-1 levels. Arginine supplementation is closely associated with IGF-1 secretion [39,40], which suggests that the addition of arginine in KDTCa may be the cause of this phenomenon, but its effect requires the combined action of calcium. We propose that the observed increase in rat long bone length is mediated through the stimulation of IGF-1 secretion, which results from the combined action of nutrients, including calcium citrate and bamboo shoot extract.

Long bone growth occurs through endochondral ossification of growth plate cells, a critical process in bone development, regulated by IGF-1 [41,42]. Our findings indicate that varying doses of KDTCa notably enhanced the heights of both the resting and proliferative zones within the growth plate, leading to an overall increase in its total height (Figure 7B–D). In contrast, growth plate height remained unaffected by the sole application of calcium citrate and KDT. Based on the detection results of IGF-1 and the growth plate, KDTCa promote the release of IGF-1 by affecting the GH–IGF axis, thereby influencing growth plate cells. This leads to an increase in resting zone cells and stimulates the proliferation of chondrocytes, promoting endochondral ossification. Furthermore, mesenchymal cells are closely associated with the formation of growth plates, as they aggregate through adhesion molecules to form condensates, which is a prerequisite for growth plate development [43]. Previous studies have confirmed that polysaccharides can promote the osteogenic differentiation and proliferation of mesenchymal cells [44,45]. Therefore, we propose that the high polysaccharide content (27.28%) in bamboo shoot extract is one of the key factors contributing to the significant increase in growth plate height.

Interestingly, our findings revealed that KDT administration alone failed to elicit any therapeutic effects on all the aforementioned parameters, demonstrating that the therapeutic efficacy of KDTCa is mediated through the synergistic interaction between calcium and KDT components, rather than through their independent actions.

The results of calcium absorption (Figure 8) showed that the LCM group had the highest apparent calcium absorption rate, which was due to long-term insufficient calcium intake [46]. However, this high absorption is not meaningful. Under the same calcium intake, the L-KDTCa group exhibited a slightly better calcium absorption rate than the CC group, suggesting a positive effect on calcium absorption (*p* > 0.05). Wang et al. found that [47] L-aspartic acid chelated calcium from oyster shell (ACOS) significantly resulted in a higher apparent calcium absorption rate. A study on calcium-binding peptides in the hydrolysates of tilapia fish phosphoproteins demonstrated that they could enhance the bioavailability of calcium [48]. Upon hydrolysis, KDTCa is enriched with amino acids that facilitate peptide-calcium binding, the content of which in the peptides represents the calcium-binding ability. In summary, we speculate that KDTCa may improve calcium bioavailability by reducing calcium excretion in feces, but the exact mechanism requires further investigation.

The hydrolysate of bamboo shoot extract contains various amino acids, including but not limited to Glu, Arg, and Asp (Table 1). Previous animal studies have confirmed that glutamic acid and alanine exhibit bone-protective effects [49,50]. Bamboo shoot extract, which may contain polypeptides comprising amino acids such as Lys, Glu, and Asp prior to hydrolysis, could play a pivotal role in the binding and utilization of calcium, as these amino acids are crucial for calcium-binding capacity [29]. Hu et al. found that the high calcium-binding capacity of SBPHs may be directly related to the involvement of amino groups in arginine and lysine in calcium ion binding [51]. The present study showed that dietary supplement Kidtal, containing bamboo shoot extract, is a promising compound dietary supplement for the prevention or treatment of stunting caused by malnutrition when combined with calcium supplementation, which may open a new path for the treatment of stunted children.

However, the inherent complexity of bamboo shoot extracts, attributed to their rich composition of bioactive compounds such as polysaccharides, dietary fiber, and amino acids, poses significant challenges for mechanistic studies. This study has not yet pinpointed the key components mediating bone metabolism regulation. Further compositional profiling and identification of bioactive constituents responsible for these effects remain imperative.

## 5. Conclusions

As a component in Kidtal, bamboo shoot extract contains a variety of nutrients that are beneficial for bone health, and a dietary supplement combination containing bamboo shoot extract demonstrated ameliorative effects on growth retardation in calcium-deficient adolescent rats. These findings suggest that bamboo shoot extracts may improve bone health through Promote the growth of long bones, inhibit bone resorption, and restoration of bone microarchitecture. Mechanistically, the observed promotion of longitudinal bone growth could be mediated via IGF-1-stimulated endochondral ossification in growth plate cartilage.

However, neither Kidtal alone nor calcium supplementation alone promoted IGF-1 secretion compared to the combination therapy. Further in vivo and cellular studies are required to elucidate the underlying synergistic mechanisms between these treatments, including which specific components contribute to the observed interaction.

## Figures and Tables

**Table 1:** The composition of hydrolyzed amino acids in bamboo shoot extract.

Name of Amino Acid	Content (mg/g)
Asp	6.283
Thr	1.624
Ser	1.990
Glu	6.218
Gly	1.502
Ala	2.958
Cys	0.661
Val	2.209
Met	0.571
Ile	1.888
Leu	1.816
Tyr	1.376
Phe	1.551
His	0.478
Lys	1.847
Arg	0.996
Total	33.968

**Table 2:** Nutritional components of bamboo shoot extract.

Microelement	Content
Total polysaccharides	27.28%
Total proteins	4.661%
Amino acid content	3.397%

**Table 3:** Trace elements contained in bamboo shoot extracts.

Microelement	Content (mg/kg)
Na	13.401
Mg	2.212
Al	10.077
K	2646.339
Mn	2.117
Fe	14.374
Cu	9.421
Zn	1.977
Ca	31.828

## Data Availability

Data will be made available on request as they form part of an ongoing study.

## Abbreviations

The following abbreviations are used in this manuscript:

Ala	alanine
Arg	arginine
Asp	aspartic acid
BALP	Bone-Specific Alkaline Phosphatase
BMD	bone mineral density
KDTCA	bamboo shoot extract + amino acids + calcium
KDT	bamboo shoot extract + amino acids
BV	bone volume
CC	calcium citrate
Cys	cysteine
GH	growth hormone
Glu	glutamic acid
Gly	glycine
His	histidine
ICP-MS	Inductively Coupled Plasma Mass Spectrometry
IGF-1	Insulin-like Growth Factor 1
Ile	isoleucine
LCM	low-calcium model
Leu	leucine
Lys	lysine
Met	methionine
NC	normal calcium
Phe	phenylalanine
Ser	serine
Tb. N	trabecular number
Tb. Sq	trabecular separation
Tb. Th	trabecular thickness
Thr	threonine
TRACP	Tartrate-Resistant Acid Phosphatase
Tyr	tyrosine
Val	valine
